# Preoperative Serum Metabolites and Potential Biomarkers for Perioperative Cognitive Decline in Elderly Patients

**DOI:** 10.3389/fpsyt.2021.665097

**Published:** 2021-05-20

**Authors:** Wenbin Lu, Zhengyu Jiang, Jie Huang, Jinjun Bian, Xiya Yu

**Affiliations:** ^1^Faculty of Anesthesiology, Changhai Hospital, Naval Medical University, Shanghai, China; ^2^Department of Anesthesiology, Naval Medical Center, Naval Medical University, Shanghai, China

**Keywords:** post-operative cognitive decline, metabolomics, anesthesia, biomarkers, geriatric

## Abstract

Perioperative cognitive decline is one of the perioperative neurocognitive disorders common to see in elderly patients. Although POCD increases patient mortality and hospitalization time, the exact inflammatory and related mechanisms are still unknown. Besides, the diagnosis of POCD lacks a unified and straightforward evaluation neuropsychological scale. Metabolites could reveal chemical fingerprints left behind by the cellular process, which provides a new aspect to understand the biological process behind. According to the post-operative MMSE score, 56 patients who received elective orthopedics surgery were included and divided into POCD and Non-POCD groups. Preoperative serum metabolites in both groups and post-operative serum metabolites were analyzed. We then performed an SVM model using 10 differential metabolites in preoperative samples as features to evaluate the patients' risk of POCD, which appeared to be positively associated with POCD and could be a potential biomarker. We also analyzed differential serum metabolites from preoperative and post-operative samples of POCD patients. By analyzing their overlap differential metabolites with between POCD and Non-POCD patients, we further inferred seven metabolites positively related to the POCD mechanism. Our results provide a more convenient method to aid POCD diagnosis and prevention using biomarkers and explore the possible mechanism behind.

## Introduction

Perioperative cognitive dysfunction (POCD) is an overarching term for cognitive impairment identified in the preoperative or post-operative period. This includes cognitive decline diagnosed before operation (described as neurocognitive disorder), any form of the acute event (post-operative delirium), cognitive decline diagnosed up to 30 days after the procedure (delayed neurocognitive recovery), and up to 12 months (post-operative neurocognitive disorder) (1). POCD refers to disorders affecting orientation, attention, perception, consciousness, and judgment that develop after surgery. Patients over the age of 65 who underwent non-cardiac surgery had a 26% prevalence of POCD within a few weeks, which decreased to 10% 3 months postoperatively (2). Long-term POCD prolongs patients' hospitalization time with high medical costs and increases mortality before or after discharge. POCD has become a severe medical problem and a social problem that cannot be ignored.

Metabolomics is an efficient technique used to detect perturbations in the metabolome, providing detailed information on an organism's biochemical phenotype in a healthy or pathophysiologic state (3). Metabolomics has a powerful ability to reveal the unique chemical fingerprints left behind by cellular processes (4). Metabolomics platforms could be used to track biochemical alterations that might precede or precipitate pathological changes. The metabolomics could also aid in developing diagnostic markers of PND screening, early detection, and further providing a basis for disease prevention and treatment.

Currently, the diagnosis of POCD mainly relies on the neuropsychological scale, but the scale varies in a different place, and the diagnosed process is complicated. This process needs to be supervised by professions, which turned out to be laborious and time-consuming. However, studies have reported that the level of inflammatory factors in patients may be related to the occurrence of POCD, while the exact inflammatory factors and related mechanisms remain unknown (5, 6). Recent studies have reported that multiple metabolites are associated with post-operative delirium (POD) in elderly patients, and these metabolic abnormalities could increase the fragility of the brain and then contribute to POD (7). However, whether it can serve as a predictor for POCD in elderly patients undergoing Orthopedic surgery remains unclear. Therefore, this study aimed to identify potential predictors for POCD in elderly patients with Orthopedic surgery and explore possible molecular mechanisms.

## Materials and Methods

### Study Population and Setting

We selected 56 aged patients who received elective orthopedics surgery in the Faculty of Anesthesiology of Changhai Hospital between December 2018 and May 2019 to conduct a prospective clinical study, POCD-aged study (NCT03765840). The study was approved by the Shanghai Changhai Hospital Ethics Committee (Shanghai, China) (CHEC2018-133), and written informed consent was obtained from all participants.

Patients received spinal anesthesia using an Isobaric Solution of 0.75% Bupivacaine and post-operative patient control analgesia (PCA) with Butorphanol Tartrate Injection, Flurbiprofen Axetil injection, and Fentanyl Citrate Injection without contraindication. Patients with more than 65 years old, orthopedics, American Society of Anesthesiologists (ASA) physical status I-III, consent information, the educational level above primary school, a negative Clock drawing experiment, and MMSE score of above or equal to 22 points were included. Patients with more than 85 years old, a history of neurological disorders, stroke, other significant central nervous system disease or severe psychiatric illness, multiple trauma and the presence of head injury, history of an endocrine or metabolic disorder were excluded. Blood samples with severe hemolysis and loss to follow-up were rejected.

### Assessment of POCD and Patient Grouping

Cognitive function was determined by the MMSE score at PREOP, POD1, and POD3. The reduction of the MMSE score of above to 2 points on the 3rd post-operative day compared with the preoperative day was considered evidence of cognitive decline (8).

### Blood Sample Collection

Blood samples were performed on all participants at their timepoints: preoperative sample (PREOP), post-operative day 1 (POD1), and day 3 (POD3). Blood was collected into heparinized tubes. The serum was then separated and stored in a −80°C freezer before the metabolomics analysis.

### LC/MS Analysis

Liquid chromatography-mass spectrometry analysis was carried on LC-MS (Thermo, Ultimate 3000LC, Q Exactive) platform. The parameter for both ESI+ and ESI− ion mode is listed below Heater Temp 300°C; Sheath Gas Flow rate, 45arb; Aux Gas Flow Rate, 15 arbs; Sweep Gas Flow Rate, 1arb; spray voltage, 3.0KV; Capillary Temp, 350°C; S-Lens RF Level, 30%. The data was performed with feature extraction and preprocessed with Compound Discoverer software (Thermo). Two thousand fifteen features at (ESI+) ion mode and 1,601 features at (ESI−) ion mode in this experiment, the data after editing were performed Multivariate Analysis (MVA) using SIMCA-P software (Umetrics AB, Umea, Sweden).

### Biomarker Identification

We built a supervised learning model by support vector machine (SVM) to classify POCD and Non-POCD groups. Finally, we used radial as SVM kernel function under type C classification and picked out features to construct the final SVM model. Under this parameter, the predictive accuracy was calculated.

## Results

### Basic Characteristics of POCD and Non-POCD Groups

We collected all patients' demographic data in Table 1. Fifteen out of 56 patients who received elective orthopedics surgery came up with post-operative cognitive decline and were defined as the POCD group. The rest consists of the Non-POCD group. No significant difference existed in age, gender, body mass index (BMI), ASA score, education level, preoperative MMSE score, operation duration, treatment during surgery, disease history, or other general characteristics between POCD and Non-POCD groups (two-tailed unpaired Student's *t*-test, *p* > 0.05).

**Table 1:** Baseline of the study population.

**Characteristics**	**POCD group**	**Non-POCD group**	***P*-value**
	**(*n* = 29)**	**(*n* = 60)**	
Age (y)	74.27 ± 7.64	72.90 ± 5.74	0.48
Female	6	29	0.27
ASA (I/II/III)	5/9/1	8/32/1	0.42
BMI	24.27 ± 2.49	25.63 ± 3.14	0.13
Educational (y)	12.2 ± 5.45	13.05 ± 4.82	0.58
Preoperative MMSE scores	27.93 ± 0.96	27.20 ± 1.74	0.13
Operation duration (min)	1.6 ± 0.65	1.84 ± 0.71	0.25
**Treatment during surgery**
Imidazole	0	1	0.60
Dexpemsodlac	2	9	0.42
**Disease history**
Diabetes	2	8	0.44
Hypertension	6	27	0.19
Heart disease	4	4	0.52
Hyperlipemia	0	7	0.49
Lumbar anestheisa	14	40	0.59

*ASA, American Society of Anesthesiologists; BMI, body mass index; MMSE, Mini-Mental State Examination; POCD, postoperative cognitive decline*.

### Overall Metabolomics Analysis of Blood Samples

**Figure 1:**
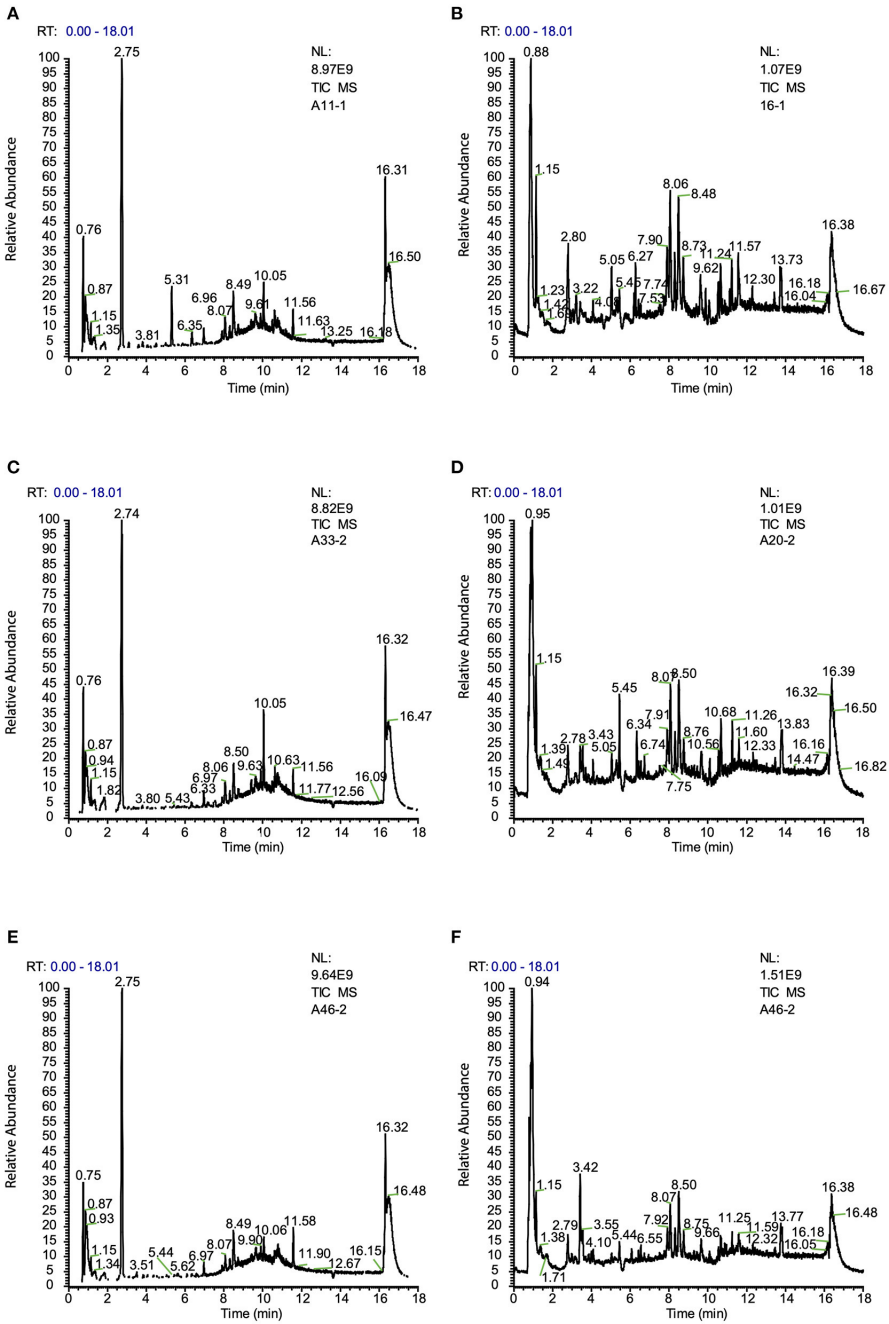
**(A)** Typical ion chromatogram from preoperative sample of Non-POCD group (Non-POCD pre-op) (ESI+). **(B)** Typical ion chromatogram from preoperative sample of Non-POCD group (ESI−). **(C)** Typical ion chromatogram from preoperative sample of POCD group (POCD pre-op) (ESI+). **(D)** Typical ion chromatogram from preoperative sample of POCD group (ESI−). **(E)** Typical ion chromatogram from post-operative sample of POCD group (POCD post-op) (ESI+). **(F)** Typical ion chromatogram from post-operative sample of POCD group (ESI−).

**Figure 2:**
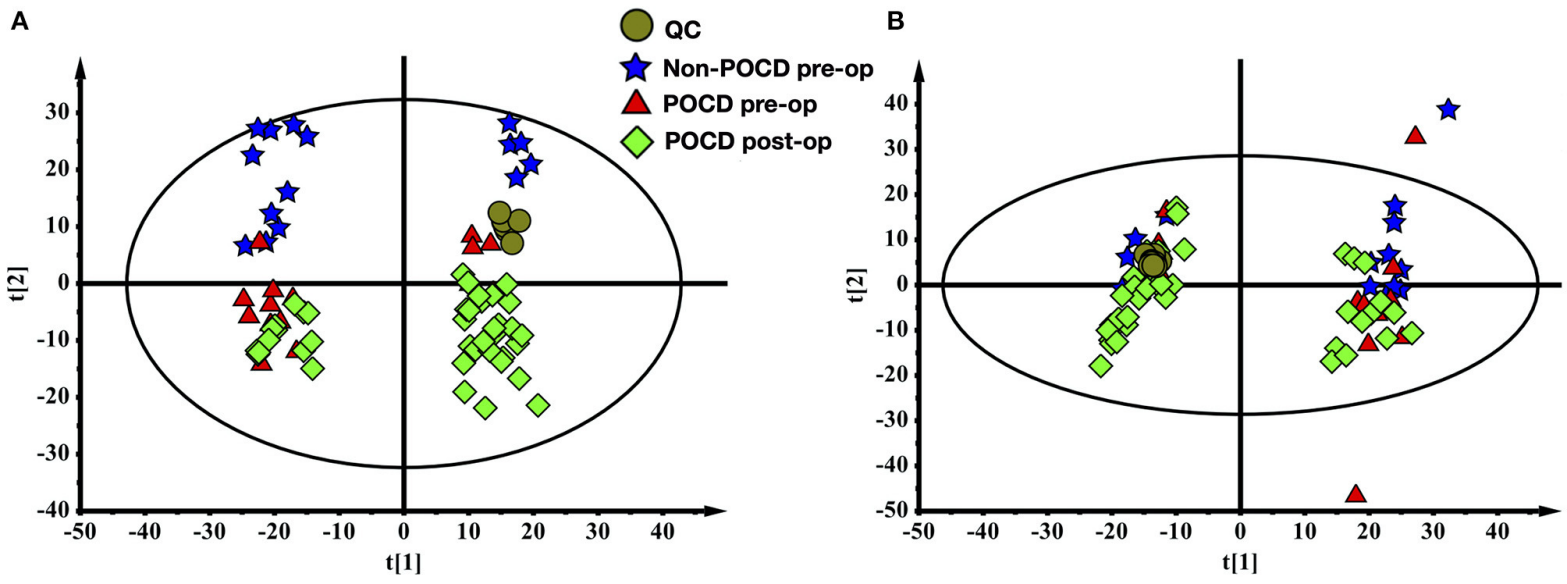
**(A)** The PCA scores plot of all samples (ESI+). **(B)** The PCA scores plot of all samples (ESI-).

The metabolomes of 71 blood samples from 56 elective orthopedic surgery patients in POCD and Non-POCD groups were characterized and compared. We collected preoperative and post-operative blood samples of all 15 patients belongs to the POCD group, while for those in the Non-POCD group, only preoperative blood samples were collected. In order to better understanding how metabolomics varied across time in POCD and Non-POCD groups, we divided them into three groups and named preoperative Non-POCD as Non-POCD pre-op, preoperative POCD group as POCD pre-op, and post-operative POCD group as POCD post-op. Representative ESI positive and ESI negative ion chromatogram of each group were presented in Figure 1. A total of 2,015 molecular features at (ESI+) ion mode and 1,601 molecular features at (ESI−) ion mode were obtained using Compound Discover and subjected to Multivariate Analysis (MVA) using SIMCA-P software. The Principal Components Analysis (PCA) extracted 11 principal components at (ESI+) ion mode with a *Q*^2^ of 0.193 and *R*^2^X of 0.504. At (ESI−) ion mode (Figure 2A), there are 12 principal components at last, with a *Q*^2^ of 0.237 and *R*^2^X of 0.579 (Figure 2B). Although at the overall point of view, these three groups' distributions were overlapped at (ESI+) ion mode and (ESI−) ion mode, the PLS-DA results showed a significant difference existed not only in between preoperative and post-operative POCD group, but also preoperative POCD and Non-POCD groups.

**Figure 3:**
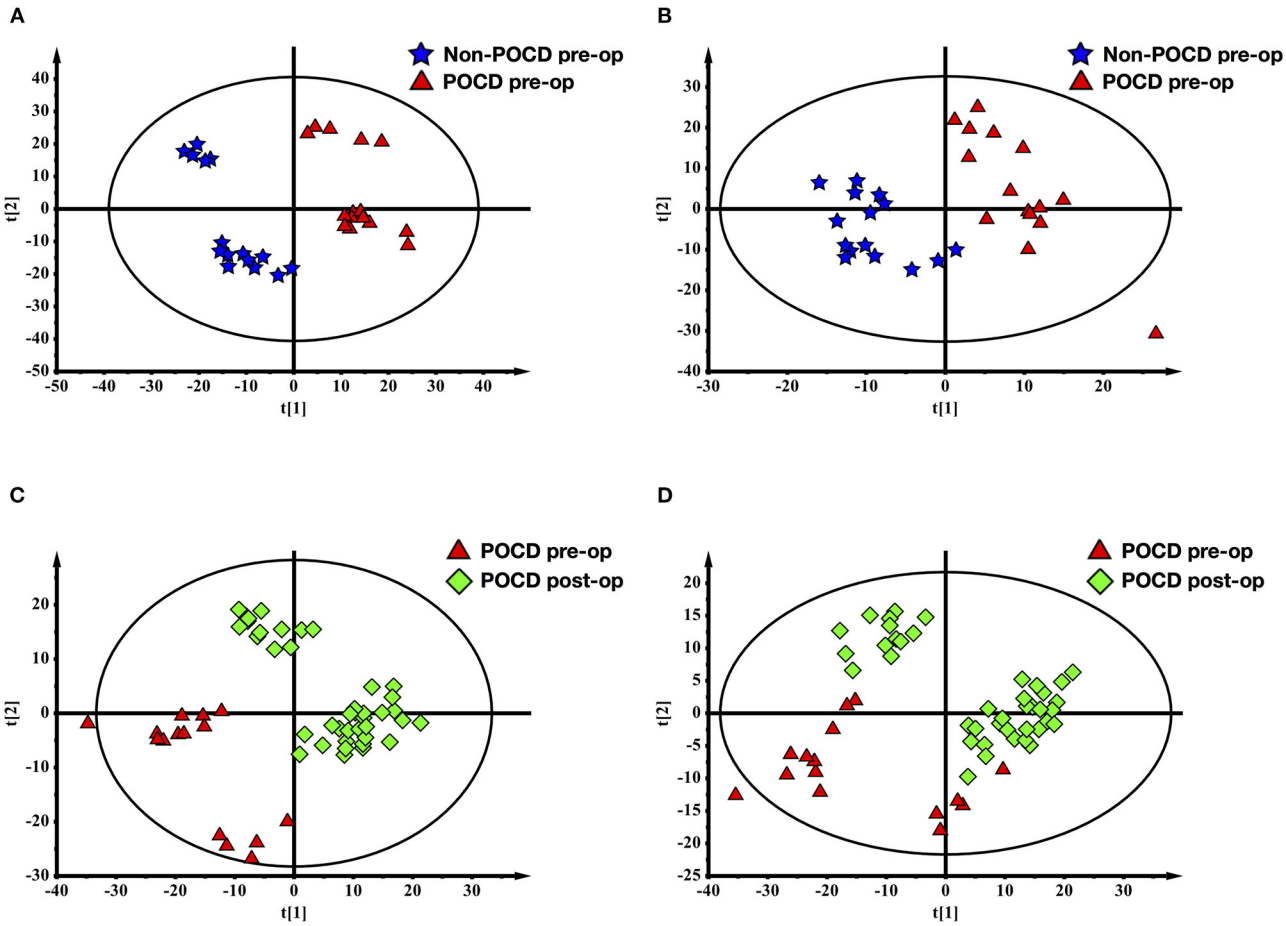
Partial least squares discriminant analysis (PLS-DA) of blood metabolomics data from before and after surgery POCD groups. Blood metabolites distinguished before and after surgery in patients with POCD. The blue stars represented before surgery POCD patients and the red triangles represented after surgery POCD patients in the two-dimensional PLS-DA score plots.

**Figure 4:**
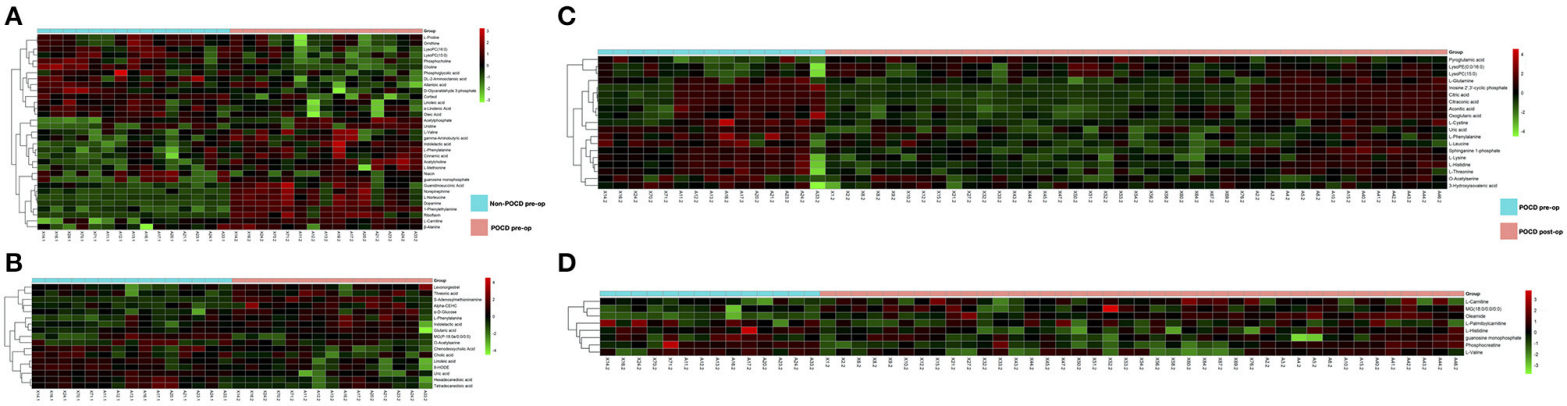
**(C,D)** Metabolic patterns in before and after surgery POCD groups. Differentiating blood metabolites between before and after surgery POCD groups were shown as heatmaps. **(A,B)** Metabolic patterns in POCD and Non-POCD groups. Differentiating blood metabolites between POCD and Non-POCD groups were shown as heatmaps. Each row represented data for a specific metabolite, and each column represented an individual. Different colors corresponded to the different intensity levels of metabolites. Red and green colors represented increased and decreased levels of metabolites, respectively.

The PLS-DA results of preoperative Non-POCD and POCD samples revealed their distributions differences with a *Q*^2^ of 0.85 and *R*^2^Y of 0.988 at (ESI+) ion mode (Figure 3A). While at (ESI−) ion mode, there is a *Q*^2^ of 0.606 and *R*^2^Y of 0.938 (Figure 3B). All the above indicated a reliable and not over lifting model. Features with VIP scores >1.0 in multivariate statistical analysis and p < 0.05 in univariate analysis were considered the most significant metabolites and were visualized through heatmap (Figures 4A,B). A total of 33 metabolites at (ESI+) ion mode and 17 metabolites at (ESI−) ion mode were picked out as the most significant differentiating metabolites (Table 2).

**Table 2:** Differential Metabolites between preoperative samples of non-POCD and POCD group.

**ESI mode**	**Name**	**VIP**	***T*-test**	**Fold change**
–	Hexadecanedioic acid	2.14886	0.00465762	−0.8624628
–	Tetradecanedioic acid	1.70424	0.02916056	−1.0766372
–	MG(P-18:0e/0:0/0:0)	1.59448	0.04243084	−1.5411543
–	Chenodeoxycholic acid	1.88515	0.01475852	−1.9328806
–	Cholic acid	1.64441	0.03589656	−1.4338451
–	Uric acid	2.29138	0.00227567	−1.4412447
–	Alpha-CEHC	1.61134	0.04012514	1.92131755
–	Linoleic acid	1.76433	0.02346783	−0.4426918
–	Levonorgestrel	1.89165	0.01437968	0.26773069
–	9-HODE	1.92393	0.01261456	−0.462167
–	O-Acetylserine	1.68166	0.03156958	−2.5438367
–	Indolelactic acid	1.85978	0.01631945	0.41675733
–	α-D-Glucose	2.70394	0.00017788	0.74054927
–	L-Phenylalanine	1.69683	0.02993438	0.65260587
–	Threonic acid	1.61069	0.04021291	0.36366708
–	Glutaric acid	1.56104	0.04731478	0.36632427
–	S-Adenosylmethioninamine	1.90984	0.01336146	7.50779703
+	LysoPC(16:0)	1.48431	0.0137353	−0.3713673
+	L-Carnitine	2.29217	2.73E-05	1.21871568
+	LysoPC(15:0)	1.76318	0.00267329	−0.4518569
+	Linoleic acid	1.56821	0.00874882	−0.2922978
+	Phosphocholine	1.6305	0.0061234	−0.7786643
+	α-Linolenic acid	1.71918	0.00355768	−0.6273769
+	Guanidinosuccinic acid	1.69301	0.00419472	1.26378252
+	Oleic acid	1.2717	0.0376366	−0.3019954
+	Niacin	1.26294	0.03908499	−2.5941806
+	L-Norleucine	2.07672	0.00023863	3.34867892
+	L-Phenylalanine	1.87914	0.00118836	0.55943035
+	Acetylcholine	2.05487	0.00028955	0.52197724
+	Cortisol	1.75185	0.00288055	−1.2338256
+	Indolelactic acid	1.66467	0.00499229	0.51636442
+	L-Methionine	1.40622	0.02032011	0.48531376
+	Allantoic acid	1.2164	0.0475443	−0.443987
+	DL-2-Aminooctanoic acid	1.63423	0.00598998	−1.1246342
+	Acetylphosphate	1.23838	0.04338481	0.66150338
+	Cinnamic acid	1.57441	0.0084509	0.42270895
+	L-Proline	1.24398	0.04237309	−0.2680731
+	Ornithine	1.41773	0.01921182	−0.3135891
+	L-Valine	1.25881	0.03978378	0.30784489
+	1-Phenylethylamine	2.81303	4.90E-09	4.67708733
+	Choline	2.08327	0.000225	−0.6555473
+	D-Glyceraldehyde 3-phosphate	1.64799	0.00551984	−0.1866975
+	gamma-Aminobutyric acid	1.6039	0.00714819	0.72901899
+	Riboflavin	1.55401	0.00946474	6.61277382
+	Phosphoglycolic acid	1.21053	0.0487076	−0.5983833
+	β-Alanine	1.27363	0.03732392	0.3704572
+	Uridine	1.4227	0.01874865	0.81909974
+	Guanosine monophosphate	1.21342	0.04813268	−2.8760388
+	Norepinephrine	1.5629	0.00901071	4.36193738
+	Dopamine	2.00847	0.00043058	5.19393492

The PLS-DA results of pre- and post-operative POCD samples revealed their distributions differences with a *Q*^2^ of 0.864 and *R*^2^Y of 0.997 at (ESI+) ion mode (Figure 3C). While at (ESI−) ion mode, there is a *Q*^2^ of 0.755 and *R*^2^Y of 0.995 (Figure 3D). Features with VIP scores >1.0 in multivariate statistical analysis and *p* < 0.05 in univariate analysis were considered the most significant metabolites and were visualized through heatmap (Figures 4C,D). A total of 8 metabolites at (ESI+) ion mode and 19 metabolites at (ESI−) ion mode were picked out as the most significant differentiating metabolites (Table 3).

**Table 3:** Differential Metabolites between pre- and post-operative samples of POCD group.

**ESI mode**	**Name**	**VIP**	***T*-test**	**Fold change**
+	L-Carnitine	1.05446	0.03856454	−0.3315896
+	L-Palmitoylcarnitine	1.38893	0.02943505	0.32502421
+	Phosphocreatine	1.43812	0.01731043	0.49435006
+	MG(18:0/0:0/0:0)	1.36982	0.02039343	−0.3346002
+	Oleamide	3.08914	9.14E-09	−0.5064589
+	L-Histidine	1.59513	0.00940359	0.29416521
+	L-Valine	1.07207	0.03307673	0.6734851
+	Guanosine monophosphate	1.25575	0.02045963	1.30581288
–	Sphinganine 1-phosphate	1.33223	0.02873744	0.47631668
–	LysoPE(0:0/16:0)	1.37326	0.02396596	−0.4155295
–	LysoPC(15:0)	1.55491	0.01004277	−0.4748921
–	Inosine 2',3'-cyclic phosphate	1.22779	0.04457426	3.43857152
–	L-Cystine	1.54479	0.01057258	1.57980759
–	O-Acetylserine	1.44526	0.01720095	1.22070319
–	Citric acid	1.54659	0.01047644	1.16190283
–	Uric acid	2.01362	0.00064842	0.93337703
–	Aconitic acid	1.52169	0.0118722	0.84792544
–	L-Phenylalanine	2.36876	4.01E-05	0.74155015
–	Oxoglutaric acid	1.19973	0.04987546	0.89811137
–	L-Histidine	1.9182	0.00123145	0.72935162
–	L-Glutamine	1.32457	0.02971115	0.3023547
–	L-Lysine	1.93461	0.00110601	0.95953705
–	L-Leucine	1.79545	0.00265216	0.55727677
–	Pyroglutamic acid	1.35367	0.02615311	−0.3068578
–	Citraconic acid	1.68244	0.00509338	1.22431302
–	L-Threonine	1.93204	0.00112491	0.58191656
–	3-Hydroxyisovaleric acid	1.20647	0.04855775	0.53960927

### SVM Analysis of Before Surgery POCD and Non-POCD Group Blood Samples

**Figure 5:**
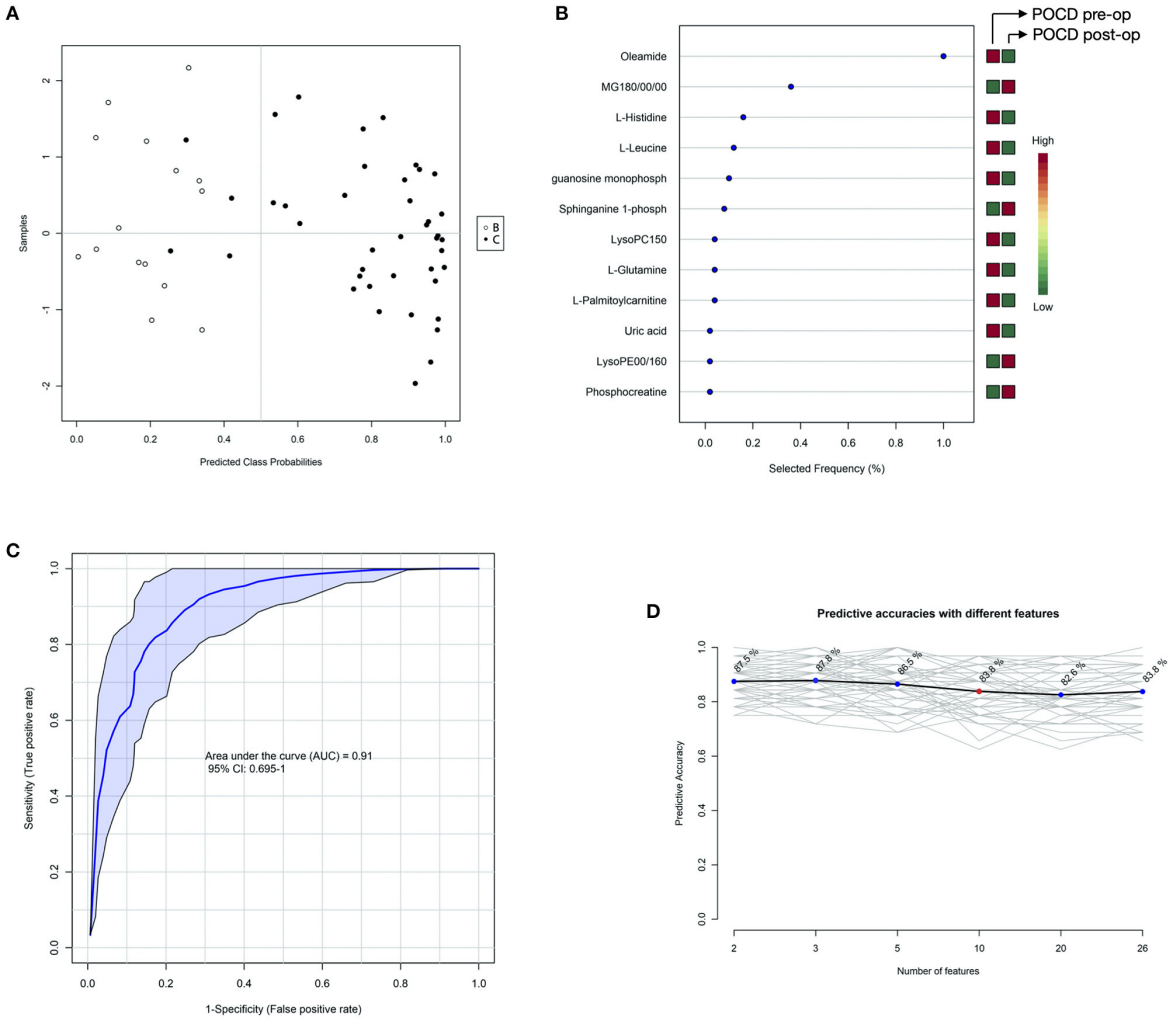
SVM analysis of before surgery POCD and Non-POCD blood samples metabolites. **(A)** SVM classification of POCD and Non-POCD groups. **(B)** Specificity and sensitivity of the SVM model. **(C)** Predictive accuracies with different features in the SVM model. **(D)** Features' importance ranking. The red cube represented higher metabolites detected while the green cube represented lower metabolites detected.

Finding out differentiating blood metabolites between preoperative samples of the POCD and Non-POCD groups inspired some of them to be used as biomarkers to predict whether a patient would suffer from POCD after surgery. Therefore, we used the support vector machine (SVM) model to identify the hidden patterns behind. Using all these differentiating blood metabolites as input features, we finally acquired an SVM model with 10 features, including oleamide, MG180/00/00, L-histidine, L-leucine, guanosine monophosphate, sphinganine 1-phospho, LysoPC150, L-glutamine, L-palmitoylcarnitine, uric acid, LysoPE00/160, and phosphocreatine (Figures 5A,B). The 0.91 AUC score indicated a well-performed model, and the prediction accuracy reaches 83.8% (Figures 5C,D). Among all these features, oleamide has the highest selected frequency and could be a potential biomarker for POCD prediction. We annotated the metabolites variation for each feature. Only oleamide, MG180/00/00, LysoPCD150, and LysoPE00/160 were higher in the Non-POCD group than the POCD group.

We also found that seven metabolites were calculated as significant differential metabolites in POCD pre-op and Non-POCD pre-op samples compared to POCD post-op samples.

## Discussion

Perioperative cognitive decline (POCD) is a post-operative complication in elderly patients, which resulted in post-operative adverse effects on orientation, attention, perception, consciousness, and judgment. Despite the severe medical burden of POCD in the perioperative period, detailed pathogenesis and molecular mechanism remain unknown. As there is limited therapy in treating the POCD, the identification of high-risk patients becomes essential. Several studies proposed that advanced age, preoperative high blood pressure and diabetes, cerebrovascular disease, and psychological factor may increase the risk of POCD. However, more sensitive and specific diagnostic methods are still in great need. By applying metabolomics, we detected significant differential metabolites between POCD and Non-POCD groups and identified 10 serum biomarkers through the support vector machine (SVM) that may act as risk factors for predicting POCD. This analysis is also applied to pre- and post-operative POCD samples to investigate possible metabolic mechanisms in developing POCD.

**Figure 6:**
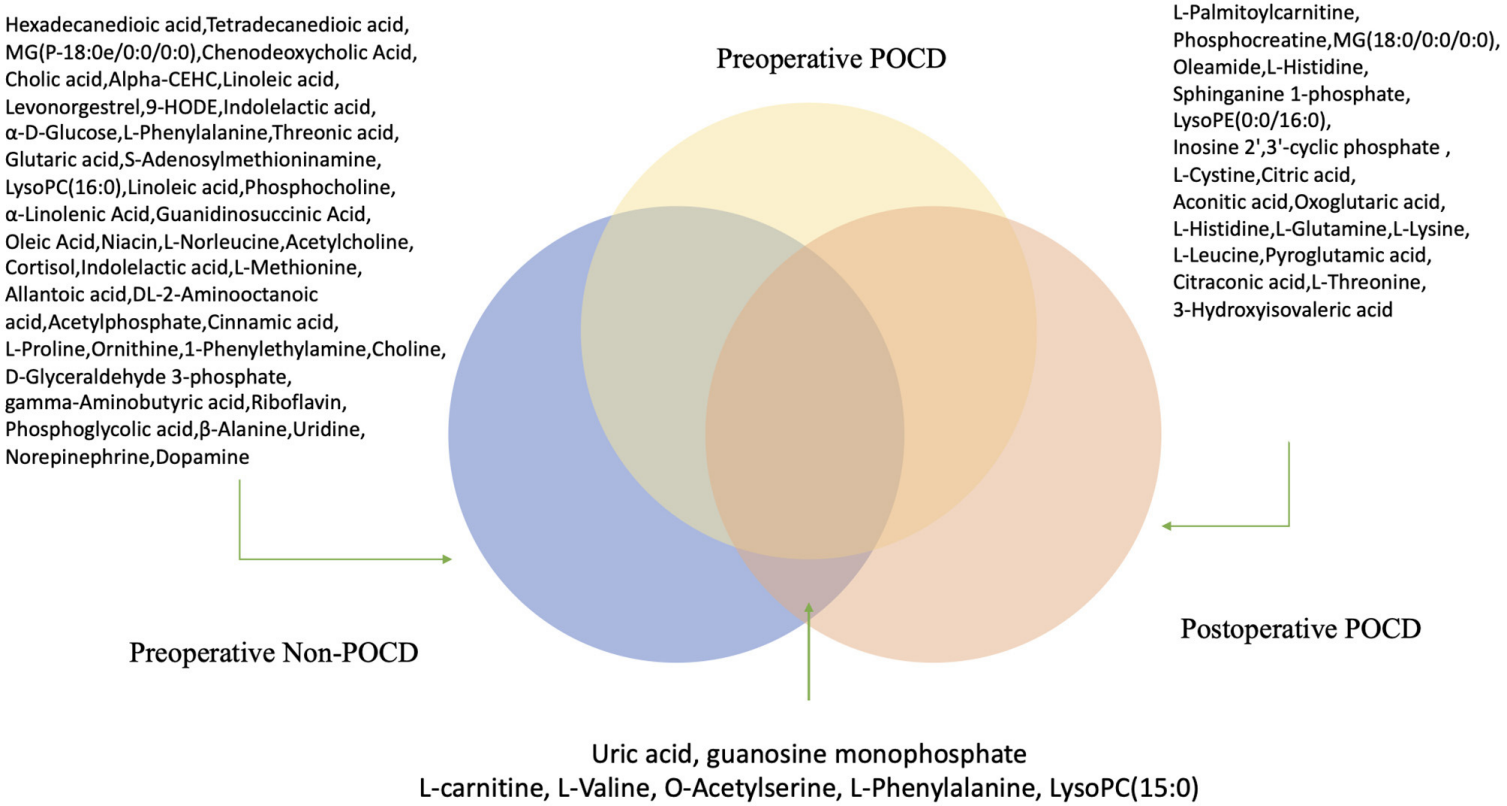
Hypothetical figure for significant differential metabolites appeared in both preoperative comparisons of POCD and Non-POCD groups, and pre- and post-operative comparison of the POCD group.

In our study, seven kinds of differential metabolites appeared in both preoperative comparisons of POCD and Non-POCD groups, and pre- and post-operative comparison of the POCD group (Figure 6). We also dig into their potential biological mechanism related to cognitive. Compared to L-valine, O-acetylserine and L-phenylalanine, LysoPC (15:0), uric acid, and guanosine monophosphate were tended to be a possible inducement of POCD. At the same time, the variation of amino acid L-valine, O-acetylserine, and L-phenylalanine are more likely to be a downstream result influenced by the system alteration. Purine metabolism was proved to have an important function in regulating gut motor reflexes and associate with inflammatory bowel disease (9). At the same time, gut microbiota, which induced neuroinflammation, might be related to cognitive dysfunction (9). These metabolites could be a pilot process of how intestinal flora influence cognitive.

Simply analysis of differential metabolites could only offer limited information. With the SVM model's help, we can predict 83.8% potential POCD patients using 10 metabolites. In this way, patients who are predicted to be at high risk of POCD may be considered to take precautionary treatments such as the perioperative application of dexmedetomidine or the avoidance of inhalation anesthetics. We can diagnose and predict the risk of a patient with a SVM model constructed using only 10 serum metabolites. And the uniform standard could be easier to measure than the evaluation based on age and accompanying disease. In previous research, oleamide, which ranked top among all 10 metabolites in our study, was proved to reverse the scopolamine-induced memory and cognitive impairment in the passive avoidance test and Y-maze test (10). Therefore, the supplement of decreased oleamide in the POCD group might be helpful for the cognitive decline.

These differential metabolites between both preoperative comparisons of POCD and Non-POCD groups, and pre- and post-operative comparison of the POCD groups indicate the difference in serum sample. It shows a way to further dig into the POCD mechanisms. The SVM model gives a uniform standard for clinical diagnose which is easier and measurable compared to traditional methods.

There are also limitations to the present study. Firstly, this is a small-sample single-center prospective study, and we only recruited patients who underwent orthopedic surgery. Selection bias and deviation in metabolic data due to similar primary illness may influence the conclusion's interpretation and generalization. Secondly, the investigation on a metabolic mechanism based on the comparison of pre- and post-operative samples of the POCD group is still preliminary and requires further study. As our results suggested, specific metabolites may participate in the pathogenesis of the POCD. Future studies with detailed observation of analysis may help identify the specific metabolic interaction that promotes the POCD.

In conclusion, our study proposed a novel method to predict and detect POCD with metabolomics. We identified ten metabolites that may indicate the high-risk patients of POCD. The research related to oleamide inspired us to study these biomarkers and found out if some of them could be used as a potential treatment. LysoPC (15:0), uric acid, guanosine monophosphate might be an entry point to study the relationship between intestinal flora and cognitive.

## Data Availability Statement

The original contributions presented in the study are included in the article/Supplementary Material, further inquiries can be directed to the corresponding author/s.

## Ethics Statement

The studies involving human participants were reviewed and approved by Shanghai Changhai Hospital Ethics Committee. The patients/participants provided their written informed consent to participate in this study. Written informed consent was obtained from the individual(s) for the publication of any potentially identifiable images or data included in this article.

## Author Contributions

WL and ZJ contributed to the patients' recruitment, statistical analysis, and mathematical modeling. JH contributed to the scientific oversight and literature analysis. XY and JB contributed to the project conception and funding collection. All authors contributed to the article and approved the submitted version.

## Conflict of Interest

The authors declare that the research was conducted in the absence of any commercial or financial relationships that could be construed as a potential conflict of interest.

## References

[1] Evered L Eckenhoff RG Ames D Bekker A Berger M Blacker D. Recommendations for the nomenclature of cognitive change associated with anaesthesia and surgery-−2018. Br J Anaesthesia. (2018) 129:872–9. 10.1097/ALN.0000000000002334 30325806

[2] Kotekar N Shenkar A Nagaraj R. Post-operative cognitive dysfunction - current preventive strategies. Clin Interven Aging. (2018) 13:2267–73. 10.2147/CIA.S133896 30519008 PMC6233864

[3] Patti GJ Oscar Y Gary S. Innovation: metabolomics: the apogee of the omics trilogy. Nat Rev Mol Cell Biol. (2012) 13:263–9. 10.1038/nrm3314 22436749 PMC3682684

[4] Nicholson JK Lindon JC. Systems biology: metabonomics. Nature. (2008) 455:1054–6. 10.1038/4551054a 18948945

[5] Androsova G Krause R Winterer G Schneider R. Biomarkers of post-operative delirium and cognitive dysfunction. Front Aging Neurosci. (2015) 7:112. 10.3389/fnagi.2015.00112 26106326 PMC4460425

[6] Skvarc DR Berk M Byrne LK Dean OM Dodd S Lewis M. Post-Operative cognitive dysfunction: an exploration of the inflammatory hypothesis and novel therapies. Neurosci Biobehav Rev. (2018) 84:116–33. 10.1016/j.neubiorev.2017.11.011 29180259

[7] Guo Y Zhang Y Jia P Wang W Zhou Q Sun L. Preoperative serum metabolites are associated with postoperative delirium in elderly hip-fracture patients. J Gerontol Series A. (2017) 72:1689–96. 10.1093/gerona/glx001 28180239

[8] Xu JH Zhang TZ Peng XF Jin CJ Zhou J Zhang YN. Effects of sevoflurane before cardiopulmonary bypass on cerebral oxygen balance and early post-operative cognitive dysfunction. Neurol Sci Official J Italian Neurol Soc Italian Soc Clin Neurophysiol. (2013) 34:2123–9. 10.1007/s10072-013-1347-3 23525738

[9] Christofi FL Bharucha AE. Gastrointestinal: new frontiers in therapeutics for diseases GI, and disorders—from microRNAs to novel pharmaceutics, to low FODMAP diets and microbiome. Curr Opin Pharmacol. (2017) 37:iv–viii. 10.1016/j.coph.2017.11.011 29224799 PMC6075818

[10] Heo HJ Park YJ Suh YM Choi SJ Kim MJ Cho HY. Effects of oleamide on choline acetyltransferase and cognitive activities. Biosci Biotechnol Biochem. (2003) 67:1284–91. 10.1271/bbb.67.1284 12843655

